# Untargeted plasma metabolite detection in sudden sensorineural hearing loss: identifying key metabolic signatures

**DOI:** 10.3389/fmolb.2025.1567064

**Published:** 2025-07-14

**Authors:** Rongyue Ma, Huangruici Zhang, Weijie Wang, Changping Yu, Guohang Xiong, Qing Li, Yan Wang, Li Zhou, Yu Zhang, Min Li, Min Guo

**Affiliations:** ^1^ Department of Otorhinolaryngology, The First Affiliated Hospital of Kunming Medical University, Kunming, Yunnan, China; ^2^ Research Center for Clinical Medicine, The First Affiliated Hospital of Kunming Medical University, Kunming, Yunnan, China; ^3^ Department of Nephrology, The First Affiliated Hospital of Kunming Medical University, Kunming, Yunnan, China

**Keywords:** sudden sensorineural hearing loss (SSNHL), plasma metabolites, LC-MS analysis, differential metabolites, metabolic pathways

## Abstract

**Background:**

Sudden sensorineural hearing loss (SSNHL) is a common otological disorder with complex etiologies and unclear pathophysiology. This study aimed to detect and analyze plasma metabolites in SSNHL, identify potential biomarkers, and uncover underlying metabolic mechanisms.

**Methods:**

A cohort of 64 SSNHL, classified into four subtypes (low-frequency, high-frequency, flat and total deafness type), and 53 normal controls (NC) were recruited. Plasma samples were collected and analyzed by high-performance liquid chromatography-mass spectrometry (LC-MS). Metabolite profiling was performed, followed by multivariate statistical analyses, including orthogonal projections to latent structures-discriminant analysis (OPLS-DA) and partial least-squares discriminant analysis (PLS-DA) to find differentially expressed metabolites between the groups.

**Results:**

The results showed significant differences in the plasma metabolome when comparing each of the four SSNHL types with NC. A total of 130 differentially expressed metabolites were identified, with sphingosine, anthranilic acid, and 6-hydroxyflavanone (6-HF) being prominent examples. Were prominent. Pathway enrichment analysis indicated that these metabolites were mainly involved in central carbon metabolism, protein digestion and absorption, aminoacyl-tRNA biosynthesis, mineral absorption, etc.

**Conclusion:**

These findings imply that plasma metabolite profiling holds promise as a non-invasive approach for screening biomarkers in SSNHL. The identified differential metabolites and associated metabolic pathways may offer novel perspectives on the pathophysiology of SSNHL, presenting potential targets for future therapeutic interventions.

## 1 Introduction

Sudden sensorineural hearing loss (SSNHL) is a common disease in otorhinolaryngology, with an annual incidence rate of (5-20)/100,000 (Cadoni et al., 2003). In recent years, the incidence rate of SSNHL in China has been increasing year by year. Due to the lack of direct pathological evidence, the etiology and pathogenesis of sudden deafness remain unclear. The theories mainly tend to focus on viral infection, inner ear microcirculation disorders, autoimmune factors, rupture of the round window membrane, peroxidation factors, psychological factors, and other aspects (Schreiber et al., 2010). Besides hearing loss, SSNHL is often accompanied by symptoms such as tinnitus and dizziness, which not only affect the quality of life of patients, but also have a certain degree of damage to their mental health. Severe cases may lead to psychological problems such as anxiety and depression. At present, domestic clinical work guidelines have divided SSNHL into low-frequency type (LD), high-frequency type (HD), flat type (FD) and total deafness type (TD) according to hearing curves. It is believed that the pathogenesis of each type is not exactly the same, and different treatment programs have been formulated accordingly.

Emerging evidence suggests, numerous studies have been conducted on the pathogenesis, early prediction, pathological changes, preventive interventions, prognostic factors, etc. Of SSNHL. Nevertheless, the precise pathophysiology remains incompletely understood, with approximately 50% of cases classified as idiopathic, and no disease-specific biomarkers currently established (Boullaud et al., 2022). Metabolomics offers a pivotal approach to systematically profile low-molecular-weight metabolites (<1,500 Da) in biological systems, capturing dynamic interactions between genetic and environmental factors (Goodacre, 2005). By operating downstream of central dogma processes in biological systems, this technology reveals real-time pathophysiological disturbances in cochlear metabolism, highlighting its potential for etiological stratification, progression monitoring, and personalized therapeutic strategies in hearing impairment (Boullaud et al., 2022).

Current metabolomic investigations of auditory dysfunction predominantly utilize three biofluid matrices: perilymph, plasma, and urine. While perilymph directly reflects cochlear pathophysiology, the invasiveness of the sampling procedure and sample contamination risks limit its clinical utility (typically yielding <1 μL of fluid) (Mavel et al., 2018; Trinh et al., 2019). Despite this challenge, Mavel et al. (2018) demonstrated the technical feasibility of generating metabolomic profiles from such samples, and Trinh et al. (2019) subsequently identified metabolite signatures correlated with hearing loss duration, highlighting the utility of perilymph. Plasma analysis necessitates standardized collection protocols (heparinized tubes, 6–8-h centrifugation before cryopreservation) (Videhult Pierre et al., 2017; Gaboon et al., 2020; Miao et al., 2021). Miao et al. (2021) documented noise-exposure associated perturbations in 20 plasma metabolites, particularly in glycerophosphate and endocannabinoid pathways. Wang et al. (2022) further identified disrupted fatty acid metabolism in SSNHL patients versus healthy controls through LC-MS-based serum analysis. Urine samples are typically first-morning collections (1 mL volume), as reported by Carta et al. (2017), Dong et al. (2013), who revealed associations between urinary 3-hydroxybutyrate/creatinine levels and glucocorticoid therapy responsiveness in SSNHL. Notably, plasma and urine demonstrate superior clinical applicability due to non-invasive collection, contrasting with the technical challenges of perilymph analysis.

Current metabolomics investigations in hearing loss predominantly employ liquid chromatography-mass spectrometry (LC-MS), gas chromatography-mass spectrometry (GC-MS), and nuclear magnetic resonance (NMR) (Malesci et al., 2023). LC-MS has emerged as the dominant platform due to its exceptional sensitivity (detecting metabolites in the picomolar-nanomolar range) and minimal sample requirements (<1 μL), making it particularly suited for analyzing trace biofluids like perilymph (Boullaud et al., 2022). In contrast, GC-MS excels in profiling volatile metabolites, while NMR offers superior structural elucidation capabilities and quantitative reproducibility (Malesci et al., 2023). Methodologically, targeted metabolomics focuses on precise quantification of predefined metabolite panels to validate hypotheses, whereas untargeted approaches enable hypothesis-free exploration of global metabolic perturbations. Both strategies have demonstrated transformative potential in oncology and neurodegenerative disease research (Malesci et al., 2023), establishing a robust methodological foundation for auditory metabolome investigations.

This study employed an LC-MS-based untargeted metabolomics approach to analyze plasma samples from 64 SSNHL patients, stratified into four subgroups based on audiometric curve configurations, to systematically characterize metabolic reprogramming patterns. By utilizing an ultra-high-performance liquid chromatography quadrupole time-of-flight mass spectrometry (UHPLC-Q-TOF/MS) platform with dual-polarity (positive/negative ion) full-scan acquisition, we comprehensively identified 1,195 metabolites. Multivariate modeling through orthogonal partial least squares-discriminant analysis (OPLS-DA) delineated intergroup metabolite disparities, while Kyoto Encyclopedia of Genes and Genomes (KEGG) pathway mapping elucidated the disturbance patterns of metabolic pathways. These analyses aimed to identify metabolite profiles associated with SSNHL and explore pathological connections between metabolites and disease mechanisms.

## 2 Methods

### 2.1 Study participants details

64 patients with SSNHL admitted to the ENT department of the First Affiliated Hospital of Kunming Medical University were selected as the study subjects. Clinical data including, but not limited to, name, age, sex, affected side, onset time, and accompanying symptoms were collected. All patients met the criteria for the diagnosis and treatment of sudden deafness formulated by the Editorial Committee of the Chinese Journal of Otolaryngology-Head and Neck Surgery and the Society of Otolaryngology-Head and Neck Surgery of the Chinese Medical Association in 2015 (Chinese Otorhinolaryngology Head and Neck Surgery Society of Chinese Medical Association, 2015) (hereinafter referred to as “Guidelines”). Relevant audiological and imaging examinations were performed to exclude vestibular aqueduct enlargement, middle-ear lesions, Meniere’s disease, retrocochlear space-occupying lesions, traumatic deafness, infectious deafness, and other diseases, as well as other complications such as stroke, nasopharyngeal malignant tumor, acoustic neuroma, and other diseases. All patients signed the informed-consent form, and the enrollment was approved by the hospital ethics committee.

53 healthy volunteers of the same period and similar age were selected as NC. The inclusion criteria for the volunteers were as follows: no history of middle-ear and inner-ear diseases (including vocal-cord polyps, epiglottic cyst, osteoma of the external auditory canal, chronic otitis media, noise exposure, ear trauma, and ototoxicant exposure), no family history of deafness, no history of major diseases and major operations, and no participation in other studies within 3 months.

For both the case group and the control group, fasting blood samples were collected from 5:00 a.m. to 6:00 a.m. on the second day after admission.

### 2.2 Untargeted metabolomics

This study utilized high-resolution non-targeted metabolomics to detect metabolites in plasma. After the samples were slowly thawed at 4°C, appropriate amounts of samples were added to a pre-cooled methanol/acetonitrile/water solution (2:2:1, v/v). The mixture was vortex-mixed, followed by low-temperature sonication for 30 min. Then, it was left standing at −20°C for 10 min, and subsequently centrifuged at 14,000 g at 4°C for 20 min. The supernatant was taken and dried under vacuum. Next, 100 μL of an acetonitrile aqueous solution (acetonitrile: water = 1:1, v/v) was added to reconstitute the sample for mass spectrometry analysis. The sample was vortex-mixed again, centrifuged at 14,000 *g* at 4 °C for 15 min, and the supernatant was taken for injection analysis. Ultra-high-performance liquid chromatography-tandem time-of-flight mass spectrometry and Orbitrap mass spectrometry were used in series. The raw data were converted to the “mzXML” format by *P*roteoWizard. Peak alignment, retention time correction, and peak area extraction were performed using XCMS software. The data extracted by XCMS were first subjected to metabolite structure identification and data pre-processing. Then, the quality of the experimental data was evaluated. Finally, the data were analyzed.

Mass spectrometer: AB Triple TOF 6600 (AB SCIEX), Exactive HF-X (Thermo) and Q Exactive HF (Thermo); Chromatograph: Agilent 1290 Infinity LC (Agilent) and Vanquish UHPLC (Thermo). Column: Waters, ACQUITY UPLC BEH Amide 1.7 μm, 2.1 mm × 100 mm column. QC samples were inserted into the sample queue for monitoring and evaluating the stability of the system and the reliability of experimental data.

### 2.3 Untargeted metabolomics data processing and compound identification workflow

Raw MS data underwent mzXML conversion via ProteoWizard MSConvert followed by XCMS-based processing in R, with sequential workflows executed: (1) Peak detection utilizing CentWave algorithm with 10 ppm m/z tolerance, 10–60 s peak width, and prefilter thresholds (S/N = 10, intensity = 100); (2) Density-based peak alignment (bw = 5 s, m/z width = 0.025 Da, minfrac = 0.5); (3) Compound Adduct Resolution Algorithm (CAMERA)-driven feature annotation for isotopic/adduct patterns; (4) Data filtration retaining features with >50% non-zero values per experimental group. Metabolite identification was performed using ±5 ppm mass accuracy criteria, while relative quantification employed XCMS-derived peak areas without internal standard normalization, consistent with untargeted metabolomics conventions.

The untargeted metabolite identification protocol integrated retention time (RT) calibration with orthogonal mass spectrometry validation: (1) Primary screening matched experimental m/z values (mass accuracy ≤10 ppm) and RT shifts (*Δ*RT ≤ 0.2 min) against the database, followed by the molecular formula prediction via adduct pattern analysis; (2) Tandem mass spectrometry (MS/MS) spectral confirmation required >60% fragment ion matches (dot-product score ≥0.6) with collision-energy-optimized reference spectra; (3) Isomeric discrimination was achieved through chromatographic behavior cross-referencing with KEGG pathway context and literature-reported biological relevance. All identifications satisfied Metabolomics Standards Initiative (MSI) Level 2 criteria, providing two-dimensional structural evidence through consensus of accurate mass (<5 ppm post-calibration), diagnostic fragments, and physicochemical retention properties.

### 2.4 Statistical analysis

After sum-normalization, the processed data were analyzed using the R package (ropls), where they were subjected to multivariate data analysis, including Pareto-scaled principal component analysis (PCA) and OPLS-DA. The 7-fold cross-validation and response permutation testing were used to evaluate the robustness of the model. The variable importance in the projection (VIP) value of each variable in the OPLS-DA model was calculated to indicate its contribution to the classification. Student’s *t*-test was applied to determine the significance of differences between two groups of independent samples. The variable importance with the VIP >1.0 and *p* value <0.05 were used to screen significantly changed metabolites. Pearson’s correlation analysis was performed to determine the correlation between variables.

## 3 Results

### 3.1 Alteration of plasma metabolites in SSNHL

**FIGURE 1:**
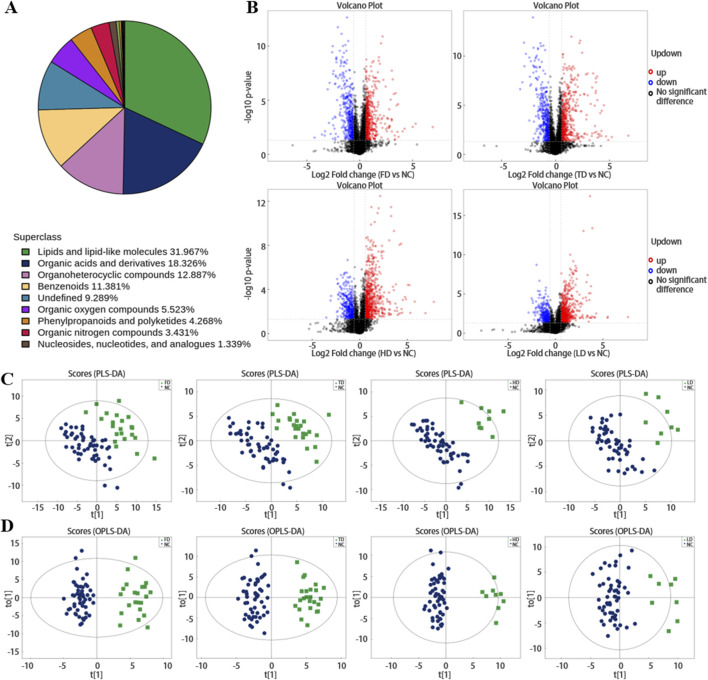
**(A)** Shows the proportion of metabolites identified by chemical classification. **(B)** Volcano plots of differential plasma metabolites in positive ion mode across FD, TD, HD and LD (red represents upregulation, blue represents downregulation, black represents non-significant differential metabolites). **(C,D)** Distributions of plasma metabolites in SSNHL.

1195 endogenous metabolites (positive: 757; negative: 438) were identified by LC-MS analysis of plasma samples. These metabolites identified by integrating both positive and negative ion data were further classified and subjected to statistical analysis based on their chemical taxonomy attribution. The proportion of each metabolite category is illustrated in Figure 1A, specifically, lipids and lipid-like molecules accounted for 31.967%, organic acids and their derivatives comprised 18.326%, Multicenter collaboration enlarges sample sizes (SSNHL patients across regions, ethnicities, subtypes) to verify metabolite universality; comparisons with other inner ear diseases (Meniere’s disease, noise-induced hearing loss) to eliminate non-specificity. Prospective cohorts compute the area under the receiver operating characteristic curve (AUC, with an ideal threshold of AUC > 0.85) for metabolite combinations (e.g., sphingosine + 6-HF), translating metabolomic discoveries into diagnostic tools like ELISA-based panels or portable MS for bedside screening compounds constituted 12.887%, and benzenoids compounds represented 11.381%. Based on univariate analysis, all metabolites (including unidentified metabolites) detected in plasma samples from the four types of SSNHL and NC were subjected to differential analysis. The results were visualized in the form of a volcano plot (for differential metabolites with fold change (FC) > 1.5 or FC < 0.05 in the positive-ion detection mode), and shown in Figure 1B; Supplementary Figure S1 (the colors of the volcano plot are used to distinguish the types of metabolites).

**FIGURE 2:**
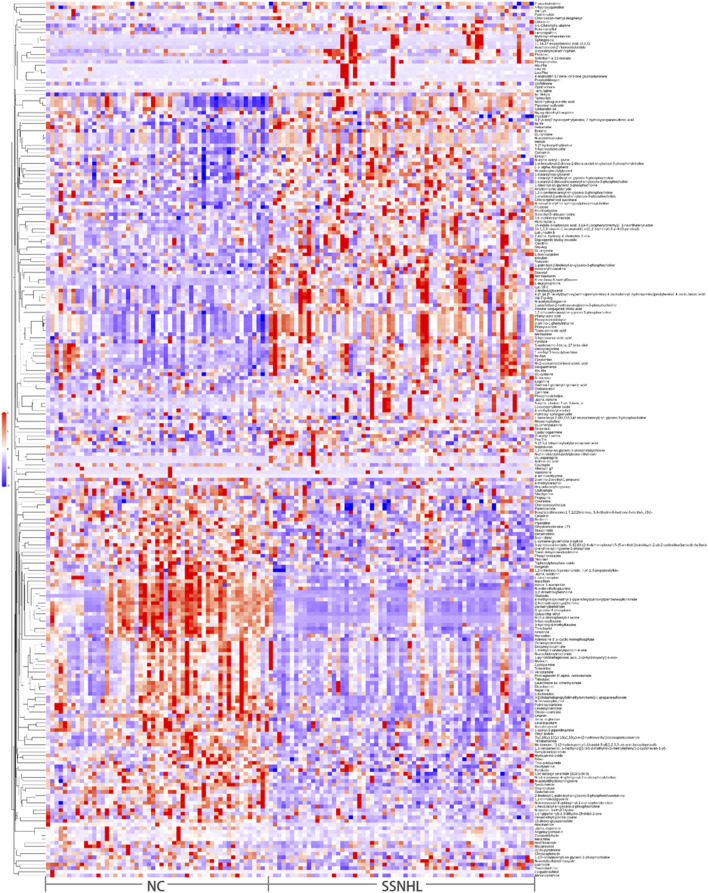
(A) Heatmap of differential plasma metabolites between SSNHL and NC, where each spot represents one sample (in positive ion mode).

Partial least squares-discriminant analysis (PLS-DA) and OPLS-DA further demonstrated that the plasma metabolome was significantly different between SSNHL and NC (both *p* < 0.05, Figures 1C,D). Using the OPLS-DA model, variables with VIP >1.0 and *p* < 0.05 were selected as criteria for screening plasma metabolites with significant differences. To present the relationships among samples and the disparities in metabolite expression patterns more comprehensively and intuitively, a distance matrix was employed to calculate the expression levels of all specimens and differential metabolites. This distance matrix utilized hierarchical clustering for clustering analysis. The analysis results of significantly different metabolites are shown in Figure 2 (between non-classified SSNHL and NC), and the results of each type of SSNHL are shown in Supplementary Figures S2A–S5A.

**FIGURE 3:**
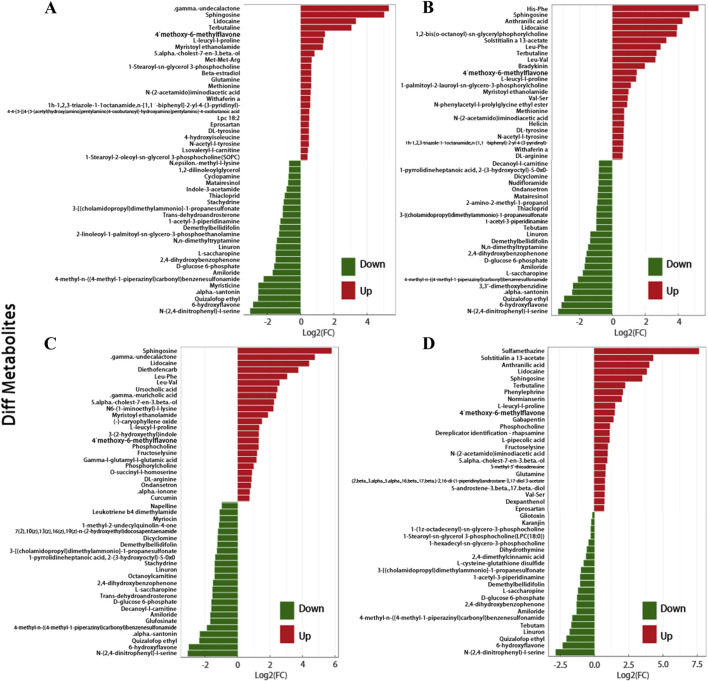
**(A)** Butterfly Plot of Fold Change. It visually represents the significant fold change differences in metabolite expression between SSNHL and NC, highlighting potential biomarkers. Respectively representing: **(A)** FD, **(B)** TD, **(C)** HD, **(D)** LD. This figure only shows the top 48 metabolites in terms of their FC values.

When comparing various types of SSNHL with the NC, the following differences were observed. In the FD group, 53 metabolites were enriched (γ-undecalactone most significant) and 49 depleted (N-(2,4-dinitrophenyl)-L-serine most significant). In the TD group, 59 were enriched (His-Phe most significant) and 51 depleted (N-(2,4-dinitrophenyl)-L-serine most significant). The LD group had 57 enriched (sulfamethazine most significant) and 21 depleted (N-(2,4-dinitrophenyl)-L-serine most significant). In the HD group, 73 were enriched (sphingosine (Sph) most significant) and 41 depleted (N-(2,4-dinitrophenyl)-L-serine most significant). Across all four SSNHL types, 20 metabolites like Sph, 4′-methoxy-6-methylflavone, and L-leucyl-L-proline were elevated, while 10 plasma metabolites such as linuron, amiloride, and 2,4-dihydroxybenzophenone decreased (Figures 3A–D).

By intersecting the different metabolites detected from each type of sample and drawing a Venn diagram, as is shown in Supplementary Figure S6, 34 metabolites were common to the results obtained from the four databases.

### 3.2 Functional role and correlation of differential metabolites in SSNHL

**FIGURE 4:**
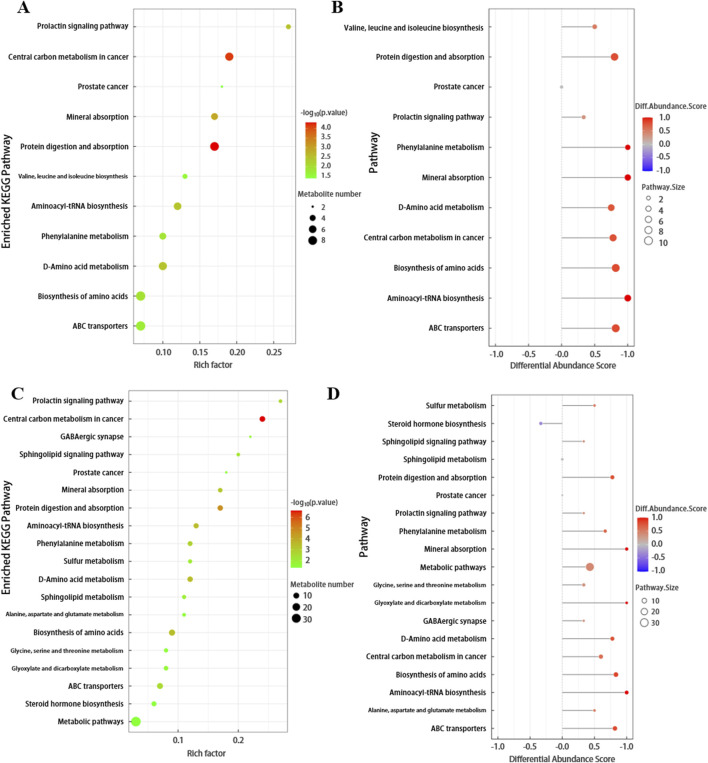
**(A)** Bubble plot of KEGG enriched pathways for FD. The x-axis represents the Rich factor, and the y-axis represents the pathway names. The size of the bubbles indicates the number of differentially expressed metabolites in each pathway, while the color of the bubbles represents the *p*-value of pathway enrichment. **(B)** Plot of Differential Abundance Scores for Enriched Metabolic Pathways. This graph displays the differential abundance scores of enriched metabolic pathways. The x-axis represents the metabolic pathways, and the y-axis represents the differential abundance scores, which reflect the degree of difference in metabolite abundances between FD and NC. **(C,D)** Bubble plot of KEGG enriched pathways and Differential Abundance Scores plot for TD.

The differential metabolites screened by positive and negative ion modes were merged and analyzed for metabolic pathways using the KEGG database (Figure 4). The pathway annotation results are detailed in Supplementary Table S1. To understand the functional significance of the altered metabolites in SSNHL, a KEGG pathway enrichment assay was performed to identify the metabolic and signal transduction pathways that were significantly affected. KEGG pathway enrichment analysis calculates the significance level of metabolite enrichment for each pathway using Fisher’s Exact Test, with the KEGG pathway as the unit and the metabolic pathways involved in this species or closely related species as the background, in order to determine the significantly affected metabolic and signal transduction pathways. As shown in Figures 4A,C; Supplementary Figures S7A, S7C, the smaller the *p*-value, the more significant the difference in the metabolic pathway, and the closer the dots are to red. Among plasma metabolites, the top differential pathways in type 4 SSNHL compared to NC were central carbon metabolism, protein digestion and absorption, aminoacyl-tRNA biosynthesis, mineral absorption, choline metabolism, and phenylalanine metabolism. The differential abundance scores of enriched metabolic pathways are displayed in Figures 4B,D; Supplementary Figures S7B, S7D (the color intensity of the dots scales proportionally with the DA score—deeper red indicates a stronger tendency toward overall upregulation of the pathway, while deeper blue indicates a stronger tendency toward overall downregulation). The expression levels of all identified metabolites in the above six metabolic pathways were up-regulated.

**FIGURE 5:**
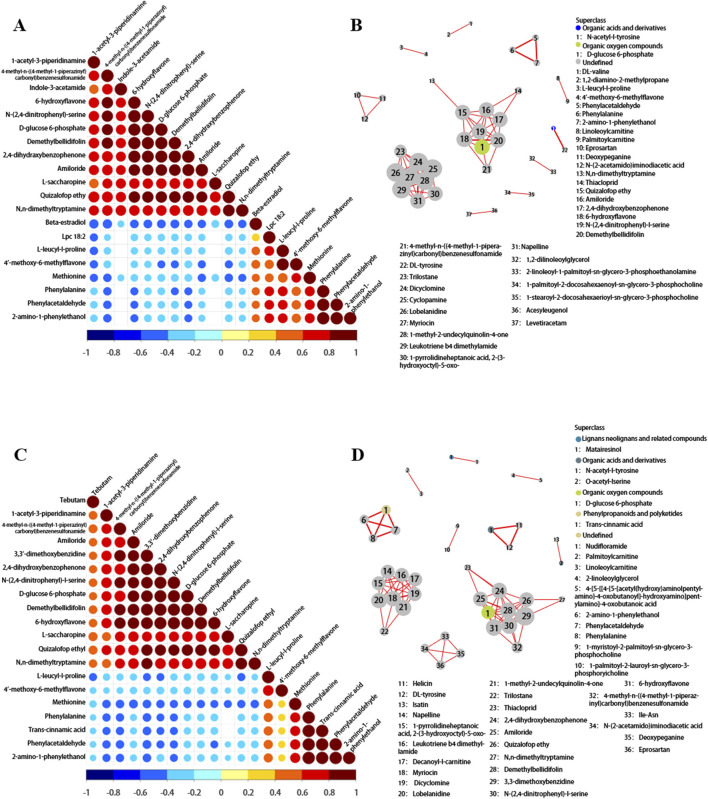
**(A)** Correlation matrix plot of metabolomics (FD). This plot is used to identify potential relationships among metabolites, which can help in understanding metabolic pathways and regulatory mechanisms. The correlation coefficients range from −1 to 1, and values closer to |1| indicate stronger correlations. **(B)** Network plot in metabolomics for FD. Nodes in the network represent metabolites, and edges represent the relationships between them. The thickness of the edges may indicate the strength of the relationships, while the color of the nodes can be used to classify different types of metabolites. **(C,D)** Correlation matrix plot and network plot of metabolomics for TD.

Correlation analysis can help measure the metabolic closeness between metabolites with significant differences, which is beneficial for further understanding the mutual regulatory relationship between metabolites in the process of biological state changes. As depicted in Figure 5A, within the plasma metabolites associated with FD, the following significant positive correlations are observed: (1) N-(2,4-dinitrophenyl)-L-serine demonstrates positive correlation with 4-methyl-N-((4-methyl-1-piperazinyl) carbonyl) benzenesulfonamide, amiloride, 3,3-dimethoxybenzidine, and 2,4-dihydroxybenzophenone. (2) Demethylbellidifolin exhibits positive correlation with 4-methyl-N-((4-methyl-1-piperazinyl) carbonyl) benzenesulfonamide, amiloride,3,3-dimethoxybenzidine, 2,4-dihydroxybenzophenone, N-(2,4-dinitrophenyl)-L-serine, and D-glucose 6-phosphate. (3) 6-hydroxyflavone reveals positive correlation with those mentioned metabolites. Moreover, there are significant negative correlations: (1) methionine correlates negatively with 1-acetyl-3-piperidinamine, 4-methyl-N-((4-methyl-1-piperazinyl) carbonyl) benzenesulfonamide, etc.; (2) phenylacetaldehyde correlates negatively with 1-acetyl-3-piperidinamine, 4-methyl-N-((4-methyl-1-piperazinyl) carbonyl) benzenesulfonamide, etc.; (3) phenylalanine correlates negatively with 1-acetyl-3-piperidinamine, 4-methyl-N-((4-methyl-1-piperazinyl) carbonyl) benzenesulfonamide, etc. Such correlations provide valuable insights into how different metabolites work together to maintain or alter biological functions.

Figure 5C shows the correlation analysis between significantly different metabolites in TD. The heatmaps of metabolite correlation in HD and LD are shown in Supplementary Figures S8A, B. The metabolic pathway association network analysis of differential metabolites between SSNHL and NC (Figures 5B,D; Supplementary Figures S9A, B) showed that differential metabolites in the metabolome were mostly associated with the metabolism of lipids and lipid-like molecules, organic acids and their derivatives, organic oxygen compounds, phenylpropanoids, and polyketides.

## 4 Discussion

The underlying pathogenesis of SSNHL remains only partially elucidated. This condition involves inner-ear microcirculation disruptions instigated by various vascular risk factors, including elevated plasma and whole-blood viscosities (Ohinata et al., 1994), pre-thrombotic gene expressions (Capaccio et al., 2009), heightened fibrinogen levels (Rudack et al., 2006), increased concentrations of blood-circulation adhesion molecules (Quaranta et al., 2008), platelet malfunction, dyslipidemia, and inflammatory responses. Viral infections can invade structures of the inner ear such as the stria vascularis and organ of Corti, damaging spiral nerve fibers and ganglion cells. Moreover, autoimmunity development, which is intricately linked to the immune regulation of the inner-ear labyrinthine organs, plays a role. Additionally, SSNHL is considered the consequence of the intricate interplay between genetic and environmental determinants. Accumulating research indicates that sleep quality, smoking habits, and certain systemic metabolic disorders, including malignant neoplasms, hypothyroidism, hypertension, diabetes mellitus, and hyperlipidemia, are also crucial risk factors contributing to the onset of SSNHL (Chau et al., 2010). Given the complex factors influencing SSNHL, blood metabolites have drawn increasing attention from scholars, as they may provide key insights into the underlying mechanisms. Thus, this study aims to explore the association between plasma metabolite changes in SSNHL and the above-mentioned factors.

Plasma metabolites were significantly altered in SSNHL, suggesting that they may contribute to the development of SSNHL. Sph and phosphocholine (PC) were enriched in all four types of SSNHL. Sph accelerates cochlear hair cell death induced by ototoxic agents (Tabuchi and Hara, 2018) and may function as an endogenous modulator mediating the apoptotic signal triggered by extracellular stimulants in some cells (Igarashi, 1997). Sph generates sphingosine 1-phosphate (S1P) through the action of Sph kinase 1 (SphK1). S1P is a pleiotropic lipid mediator that regulates cell survival and migration, immune cell recruitment, angiogenesis and lymphangiogenesis. The concentration gradient of S1P in the blood and peripheral tissues regulates lymphocyte trafficking, which is important for the pathology of inflammation and may play a role in many inflammatory diseases. Key cytokines and chemokines, such as interleukin-6 (IL-6) and tumor necrosis factor-α (TNF-α), are also linked with S1P signaling in inflammation; the S1P-Stat3-S1PR1 amplification loop also plays an important role in amplifying chronic inflammation. PDGF (platelet-derived growth factor) activates Sph kinase and causes transient S1P production in the Swiss3T3 cells. Based on this, some researchers have proposed that SlP is a mitogenic second messenger in fibroblast cell proliferation induced by PDGF and serum factors, mediates FceRI antigen receptor signaling resulting in histamine release in human mast cells (Igarashi, 1997). PC is a small lipid-related antigen. It is not only one of the important damage-associated molecular pattern molecules (DAMPs), but also a pathogen-associated molecular pattern (PAMP). Enzymatic hydrolysis and oxidative modification of phosphatidylcholine in cell membranes and low-density lipoprotein (LDL) membranes, especially fatty acids in the sn-2 position, lead to the formation of bioactive PC-containing lipids (often referred to as oxPL) (Bergmark et al., 2008). PC, the polar headgroup of oxidized phospholipids (oxPLs), is an important oxidation-specific epitope and a pro-inflammatory epitope exposed to dead cells (Caligiuri et al., 2007). In human plasma, the main carrier of oxPLs is lipoprotein a (Lp(a)), and these Lp(a)-associated oxPLs are able to induce arterial inflammation (van der Valk et al., 2016). Many of these PC-containing lipids are recognized by the innate immune system and stimulate powerful biological processes such as endothelial dysfunction, cell death, and endoplasmic reticulum (ER) stress, which are considered important mediators of vascular inflammation (de Vries et al., 2021). PC is expressed on damaged and dead cells and also on oxidized low-density lipoprotein (OxLDL). It has proinflammatory properties, when exposed on OxLDL and in other contexts (though not on apoptotic cells), these properties of PC may contribute to the immune-stimulatory and proinflammatory characteristics of OxLDL (Frostegård, 2023). Animal experiments have shown that agents that raise anti-PC levels through immunization can ameliorate atherosclerosis and other chronic inflammatory conditions, with underlying mechanisms including anti-inflammation, immunomodulation, clearance of dead cells, and protection from infectious agents (Frostegård, 2023).

Anthranilic acid was enriched in both TD and LD. Anthranilic acid is a metabolite of kynurenine produced by hosts and microorganisms. The intestinal microbiota has the function of changing the balance of anthranilic acid and its metabolites, exacerbating or causing the disease (Shaw et al., 2023). A search using the OrthoDB database revealed that six major bacterial phyla contain organisms with the kynureninase gene (KYNU), which catalyzes the conversion of kynurenine to anthranilic acid (Zdobnov et al., 2021). Anthranilic acid is a neurotoxic bioactive compound that can be used as a biomarker of acute neuroinflammation, a biomarker of depression, and even a cause. In addition, anthranilic acid can be converted to 3-hydroxyanthranilic acid by nonspecific hydroxylation, and finally metabolized to quinoline acid (QA) (Shaw et al., 2023). Elevated levels of QA can lead, acutely or chronically, to toxic effects via several mechanisms. Within the central nervous system (CNS), QA’s biological activity is primarily associated with endogenous cytotoxicity by its activation of N-methyl-D-aspartate (NMDA) receptors and mitochondrial impairment, but also involves additional targets that could be independent of its agonist activity, resulting in cellular energetic dysfunction, oxidative stress, inflammation, and cell death (Lugo-Huitrón et al., 2013). In the periphery, QA is produced in the liver, kidney, circulating monocytes, monocyte-derived macrophages (MDMs), or endothelial cells (EC) (Lovelace et al., 2016; Pawlak et al., 2002). Likewise, QA-induced toxicity in the periphery is associated with inflammation, leading to multiple organ pathologies. Other studies have shown that QA has an impact on cardiovascular disease, especially in patients with impaired renal function, and elevated QA concentrations are associated with carotid atherosclerosis (Pawlak et al., 2009a) and endothelial dysfunction (Pawlak et al., 2009b; Leszczyńska et al., 2019).

Enrichment of N-acetyl-L-tyrosine (NAT) was also observed in the plasma metabolome of TD. Fischer and Ristow (2020) identified NAT as a trigger for the excitatory effects on mitochondria in animal cells and showed that NAT helps promote the resilience of cells and organisms by inducing mitochondrial excitatory mechanisms. Other studies have shown that an increase in NAT can lead to an excess of tyrosine, which is a precursor of neurotransmitters. Excess tyrosine is converted to L-DOPA by tyrosine hydroxylase and then to dopamine, resulting in an increase in dopamine levels in the central nervous system and causing functional disorders. As a result, patients may experience symptoms such as headache, dizziness, tinnitus, and insomnia, and in severe cases, cognitive decline can occur, affecting their quality of daily life.

On the contrary, various types of SSNHL exhibited significant depletion of plasma metabolite 6-HF. 6-HF targets cyclooxygenase-2 (COX-2) and 5-lipoxygenase (5-LOX) to exert anti-inflammatory potential (Akbar et al., 2023). As a regulator of protein/enzyme function, receptor activity, and intracellular signaling, it exhibits antioxidant activity (Łodyga-Chruścińska et al., 2019). The significant consumption of 6-HF may be related to the reduction of anti-inflammatory and antioxidant cytokines, which may further exacerbate the progression of SSNHL or lead to poor prognosis for patients.

Contrastingly, a marked depletion of N, N-dimethyltryptamine (DMT) was also identified in both TD and FD. DMT is an endogenous ligand of the Sigma 1 receptor (Sig-1R), which plays various roles such as anti-apoptotic, pro-neurotrophic, anti-inflammatory, anti-hypoxic cell protective properties, and strong antidepressant and anti-anxiety effects (Cameron and Olson, 2018). Substantial consumption of decanoyl-L-carnitine was observed in both TD and HD. Decanoyl-L-carnitine is a member of the class of compounds known as acylcarnitines, which contain a fatty acid with the carboxylic acid attached to carnitine through an ester bond. Thus, decanoyl-L-carnitine is considered to be a fatty ester lipid molecule (Fındık et al., 2021).

In HD, the consumption of stachydrine (STA) was also detected. Several studies have shown that STA protects ECs against the injury induced by anoxia-reoxygenation. STA effectively reduces lipopolysaccharide (LPS)-induced endothelial inflammatory response by restraining the secretion of IL-10 and thromboxane B2 (TXB_2_). It also curbs the deleterious effect of high glucose on ECs and acted through the modulation of the SIRT1 pathway (Xie et al., 2018). STA displays potent anti-inflammatory properties by decreasing the release of key inflammatory mediators, including IL-1b and TNF-α. It also effectively blocks the phosphorylation and nuclear translocation of the NF-kB p65 subunit, thus reducing the production of inflammatory molecules. Additionally, it attenuates the interactions between platelets and neutrophils, decreasing the likelihood of inflammatory and thrombotic complications (He et al., 2024).

In FD, notable depletion of STA, indole-3-acetamide (IAM), and phosphoethanolamine was also exhibited. Studies have shown that IAM can reduce ROS production and downregulate the expression of inflammatory genes (Wang et al., 2023). Phosphoethanolamine deficiency leads to reduced phosphatidylethanolamine synthesis, which in turn affects lipid metabolism. It may increase triglyceride and fatty acid synthesis, resulting in elevated lipid levels in the blood. The consumption of L-cysteine glutathione (L-CySSG) was observed in LD. L-CySSG may be beneficial in maintaining glutathione (GSH) homeostasis and cellular antioxidant levels, preventing oxidative stress caused by GSH depletion (Berkeley et al., 2003).

In the context of metabolic reactions, alterations in metabolites indicate that they are regulated by complex pathways and networks involving various genes and proteins. Ultimately, the systemic changes in the metabolome are brought about by their interactions and mutual regulation. To investigate the physiological significance of differential metabolites in SSNHL, this study utilized the KEGG Pathway database to obtain biological information. KEGG is the most commonly used pathway database in metabolic network analysis, which is based on functional information of genes and genomes. By using metabolic reactions as clues to link possible metabolic pathways and related regulatory proteins, it displays the physiological and biochemical processes of cells. The differential metabolites in the SSNHL plasma metabolome were mostly related to metabolic pathways such as central carbon metabolism, protein digestion and absorption, aminoacyl-tRNA biosynthesis, mineral absorption, choline metabolism, and phenylalanine metabolism. Intracellular Ca^2+^ homeostasis may be a contributory factor making outer hair cells (OHCs), especially those in the high-frequency region of the cochlea, most vulnerable to environmental assault (Mammano, 2011). The auditory system consists of skeletal structures and, therefore, is prone to being affected by altered bone remodelling. Changes in bone density, mass, and dampening of the finely tuned motion mechanics of the middle ear can cause conductive hearing loss. Changes occurring in the otic capsule and the temporal bone due to demineralisation might affect the inner ear and, consequently, cause hearing loss (Singh et al., 2018). These may be the reasons why SSNHL involves mineral absorption. In addition, when phenylalanine can not be metabolized through normal pathways, like in phenylalanine hydroxylase deficiency, bypass metabolism occurs. Specifically, phenylalanine first transaminates to phenylpyruvic acid, which further metabolizes to phenylacetic acid. Then, phenylacetic acid binds with glutamine, and is catalyzed by acyltransferase in the liver to form phenylacetylglutamine. It is speculated that phenylacetylglutamine can enhance platelet activation, promote the formation of inner ear thrombosis (Nemet et al., 2020), trigger ischemia in the local microenvironment of the ear, and lead to the occurrence and aggravation of SSNHL.

The translational roadmap for SSNHL metabolomics necessitates an integrated approach bridging mechanistic discovery and clinical application. Foundational insights from multivariate modeling (OPLS-DA) and pathway analyses (KEGG) directly inform three synergistic investigative axes: 1) Mechanistic validation—Using preclinical animal models to confirm prioritized metabolites (e.g., sphingosine) causally drive cochlear dysfunction, and pairing this with therapeutic strategies like activating SphK (via FTY720 analogs/AAV gene delivery) or inhibiting SPT (with myriocin derivatives) to explore intervention pathways; 2) Multicenter collaboration enlarges sample sizes (SSNHL patients across regions, ethnicities, subtypes) to verify metabolite universality; comparisons with other inner ear diseases (Meniere’s disease, noise-induced hearing loss) to eliminate non-specificity. Prospective cohorts compute the area under the receiver operating characteristic curve (AUC, with an ideal threshold of AUC >0.85) for metabolite combinations (e.g., sphingosine + 6-HF), translating metabolomic discoveries into diagnostic tools like ELISA-based panels or portable MS for bedside screening; 3) Therapeutic innovation—conducting phase II/III RCTs to contrast metabolism - modulating regimens (e.g., anti - inflammatory metabolite supplementation) against standard care, with stratification by metabolic profiles to evaluate targeted therapeutic effectiveness. This tripartite framework systematically transitions from target identification to clinical implementation, ensuring biological relevance while addressing technical feasibility and diagnostic/therapeutic specificity.

## 5 Conclusion

In this work, a non-targeted metabolomics platform was used to reveal important plasma metabolites in SSNHL and their association with SSNHL. Multivariate statistical analysis was used to distinguish between the two groups and identify metabolites that led to separation. These different characteristic metabolites include the enrichment of metabolites produced by pro-inflammatory and pro-hypercoagulable states such as Sph and anthranilic acid, and the consumption of 6-HF, a beneficial metabolite that exerts anti-inflammatory and antioxidant activity; the metabolic pathways involved include central carbon metabolism, protein digestion and absorption, aminoacyl-tRNA biosynthesis, mineral absorption, choline metabolism, and phenylalanine metabolism. The purpose of this study is to help researchers better understand the value of metabolomic analysis for SSNHL, which will help define biomarkers associated with the cause and have important clinical significance. Metabolomic analysis has also increased understanding of the metabolic pathways involved in SSNHL and facilitated the development of new therapies. Similarly, there are limitations to this study, in which most participants were selected to avoid population heterogeneity to a large extent, but the results of this study may not be fully applicable to subjects from other populations.

## Data Availability

The data presented in our study is deposited in the Metabolomics Workbench repository, study ID ST004023, project DOI: http://dx.doi.org/10.21228/M8DZ7X (Metabolomics Workbench, 2016). Further inquiries can be directed to the corresponding author.
