# Supplementary material for: Untargeted plasma metabolite detection in sudden sensorineural hearing loss: identifying key metabolic signatures
Source: Front Mol Biosci. 2025 Jul 14;12:1567064. doi: 10.3389/fmolb.2025.1567064 (PMC12301207; doi:10.3389/fmolb.2025.1567064)
Supplement: Supplementary file 1 [file DataSheet1.pdf]

Supplementary Materials

Untargeted Plasma Metabolite Detection in Sudden Sensorineural Hearing Loss: Identifying Key Metabolic Signatures.

Rongyue Ma[1,†], Huangruici Zhang[1,†], Weijie Wang[2,†], Changping Yu[2], Guohang Xiong[2], Qing Li [3], Yan Wang[1], Li Zhou[1], Yu Zhang[1], Min Li[1], Min Guo[1,*]

[1] Department of Otorhinolaryngology, the First Affiliated Hospital of Kunming Medical University, Kunming, Yunnan, China.

[2] Research Center for Clinical Medicine, the First Affiliated Hospital of Kunming Medical University, Kunming, Yunnan, China.

[3] Department of Nephrology, the First Affiliated Hospital of Kunming Medical University, Kunming, Yunnan, China.

† These authors contributed equally to this work

* Corresponding author

E-mail addresses: guomin@kmmu.edu.cn (Min Guo)

Methods details

1. Instrument Setup and Parameter Configuration

1.1 Ultra-High Performance Liquid Chromatography (UHPLC)

Column:

Waters ACQUITY UPLC BEH Amide (1.7 μm, 2.1 mm × 100 mm), specifically designed for polar metabolite separation.

Mobile phase:

Phase A: 25 mM ammonium acetate with 25 mM ammonia aqueous solution

Phase B: Acetonitrile (HPLC grade)

Gradient elution program:

0-0.5 min: 95% B

0.5-7.0 min: 95% → 65% B (linear gradient)

7.0-8.0 min: 65% → 40% B (linear gradient)

8.0-9.0 min: isocratic 40% B

9.0-9.1 min: 40% → 95% B (linear gradient)

9.1-12.0 min: isocratic 95% B

Chromatographic conditions:

Column temperature: 25°C

Flow rate: 0.5 mL/min

Injection volume: 2 μL

1.2 Mass Spectrometry Conditions (AB Sciex TripleTOF 6600)

Ionization was performed using electrospray ionization (ESI) with alternating positive/negative polarity modes. Key parameters were set as follows:

Ion source:

Spray voltage: ±5.5 kV

Source temperature: 600°C

Gas settings:

Nebulizer gas (GS1): 60 psi

Auxiliary gas (GS2): 60 psi

Curtain gas (CUR): 30 psi

Mass analysis:

MS1 scan range: *m/z* 60-1000 (accumulation time: 200 ms)

MS2 scan range: *m/z* 25-1000 (accumulation time: 50 ms)

Collision energy: 35 ± 15 eV

Declustering potential: ±60 V

Data acquisition:

Data-dependent acquisition (DDA) with top 10 precursors per cycle

Dynamic exclusion: 4 Da window

Isotope peaks excluded

1.3 Mass Spectrometry Conditions (Thermo Scientific Q Exactive HF-X)

Parameter configuration:

Ion source:

Spray voltage: ±5.5 kV

Full MS scan:

Resolution: 60,000

Scan range: *m/z* 80-1200

Maximum injection time: 100 ms

MS/MS scan:

Resolution: 30,000

Scan range: *m/z* 70-1200

Maximum injection time: 50 ms

Data acquisition:

Dynamic exclusion window: 4 s

1.4 Supplementary Methodological Notes

Column selection rationale:

HILIC chromatography (Waters ACQUITY UPLC BEH Amide) was employed to achieve optimal retention of polar metabolites, enabling sensitive detection of plasma constituents including sphingosine and phosphocholine (logP < -1.5).

Gradient optimization:

Extended isocratic elution at 95% organic phase (acetonitrile; 9.1-12 min) enhanced hydrophobic metabolite resolution while maintaining column stability.

Data preprocessing:

Total sum normalization was applied to account for inter-sample intensity variations, thereby improving inter-group comparability through variance stabilization.

2. Sample Preparation

QC samples were prepared by mixing equal volumes of all experimental samples, with one QC sample inserted per five experimental samples.

2.1 Validation indicators:

(1) Total ion chromatograms (TIC): Chromatographic peak retention times and intensities in QC samples showed substantial overlap;

(2) Principal component analysis (PCA): QC samples clustered closely in both positive and negative ion modes, demonstrating good reproducibility;

(3) Relative standard deviation (RSD): Over 80% of ion peaks in QC samples exhibited RSD ≤30%.

2.2 Repeatability assessment:

QC samples showed correlation coefficients >0.9, and Hotelling's T^2 test confirmed all samples remained within the 99% confidence ellipse.

Inherent Limitations in Untargeted Metabolite Identification

The metabolite identification process carries inherent limitations: (1) Mass accuracy tolerance (±10 ppm) may permit misannotation of isobaric/isomeric species, compounded by instrumental drift and matrix-induced signal suppression; (2) MS/MS spectral matching reliability depends on reference database completeness and acquisition condition congruence, where divergent collision energies (±5 eV) or ionization modes between experimental and reference spectra can reduce spectral similarity scores; (3) Manual verification, while mitigating algorithmic false positives, introduces expert-dependent interpretation variability, particularly for metabolites lacking definitive literature evidence or exhibiting pathway ambiguity; (4) Database coverage gaps persist despite our curated repository of 30,000+ entries, disproportionately affecting novel metabolites and xenobiotics without authenticated standards. These uncertainties collectively underscore the tentative nature of Level 2 identifications per Metabolomics Standards Initiative guidelines.

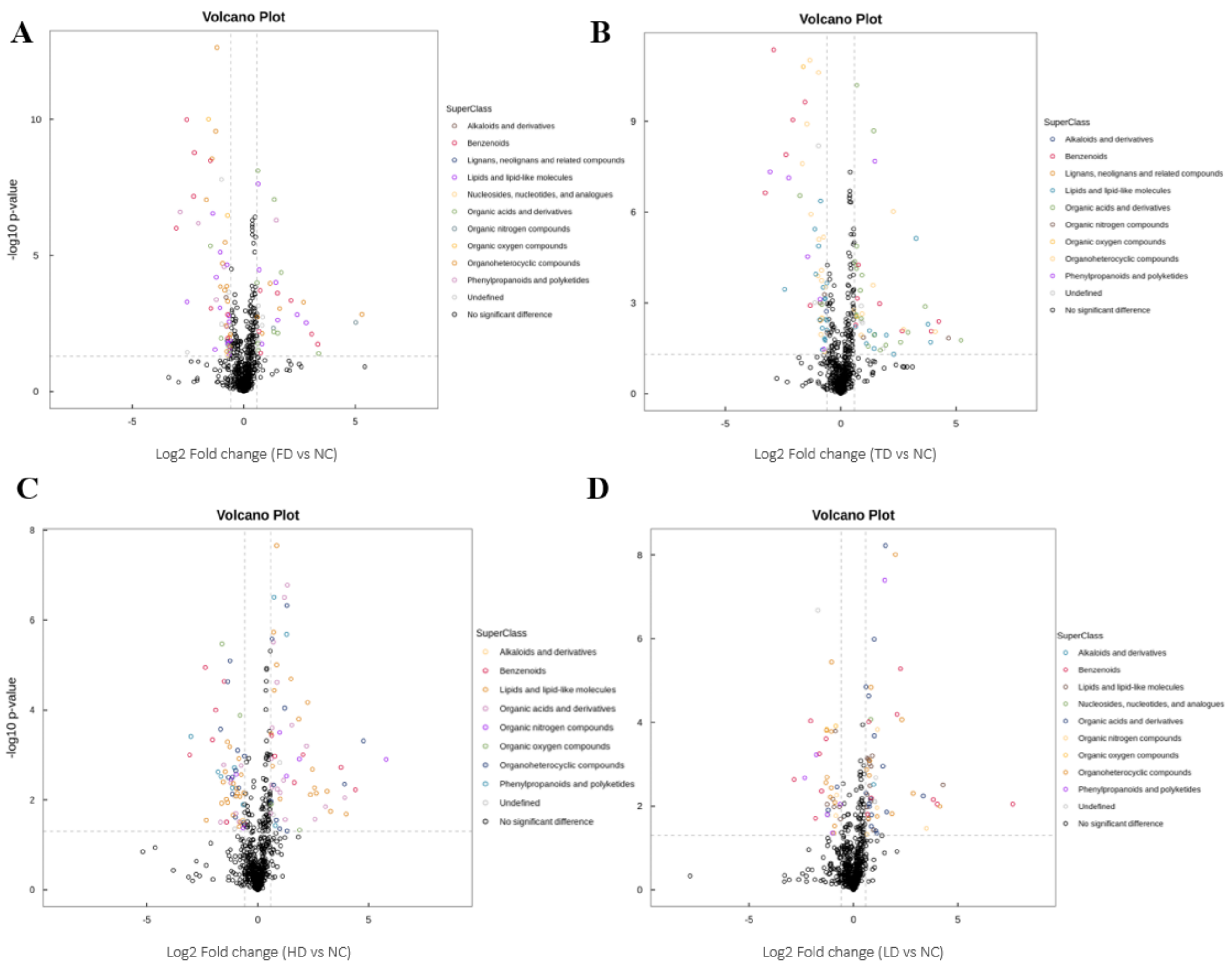

Supplementary Figure 1 (A-D) Volcano Plots in Positive Ion Mode for FD, TD, HD and LD (Colors are related to the chemical classification of differential metabolites).

A

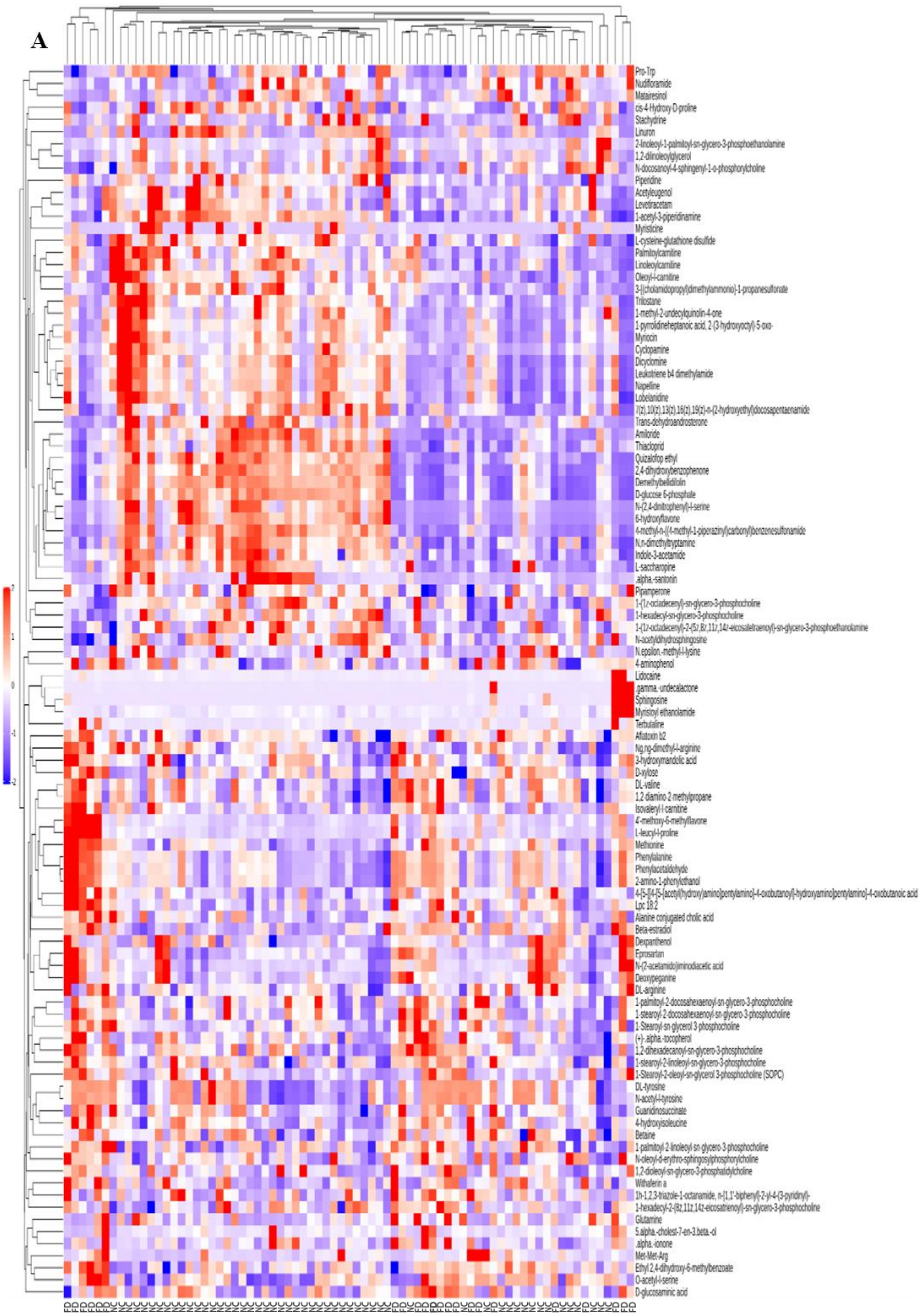

Supplementary Figure 2(A) Hierarchical Clustering Heatmap of significantly differentially expressed metabolites in FD (VIP > 1, p-value < 0.05).

A

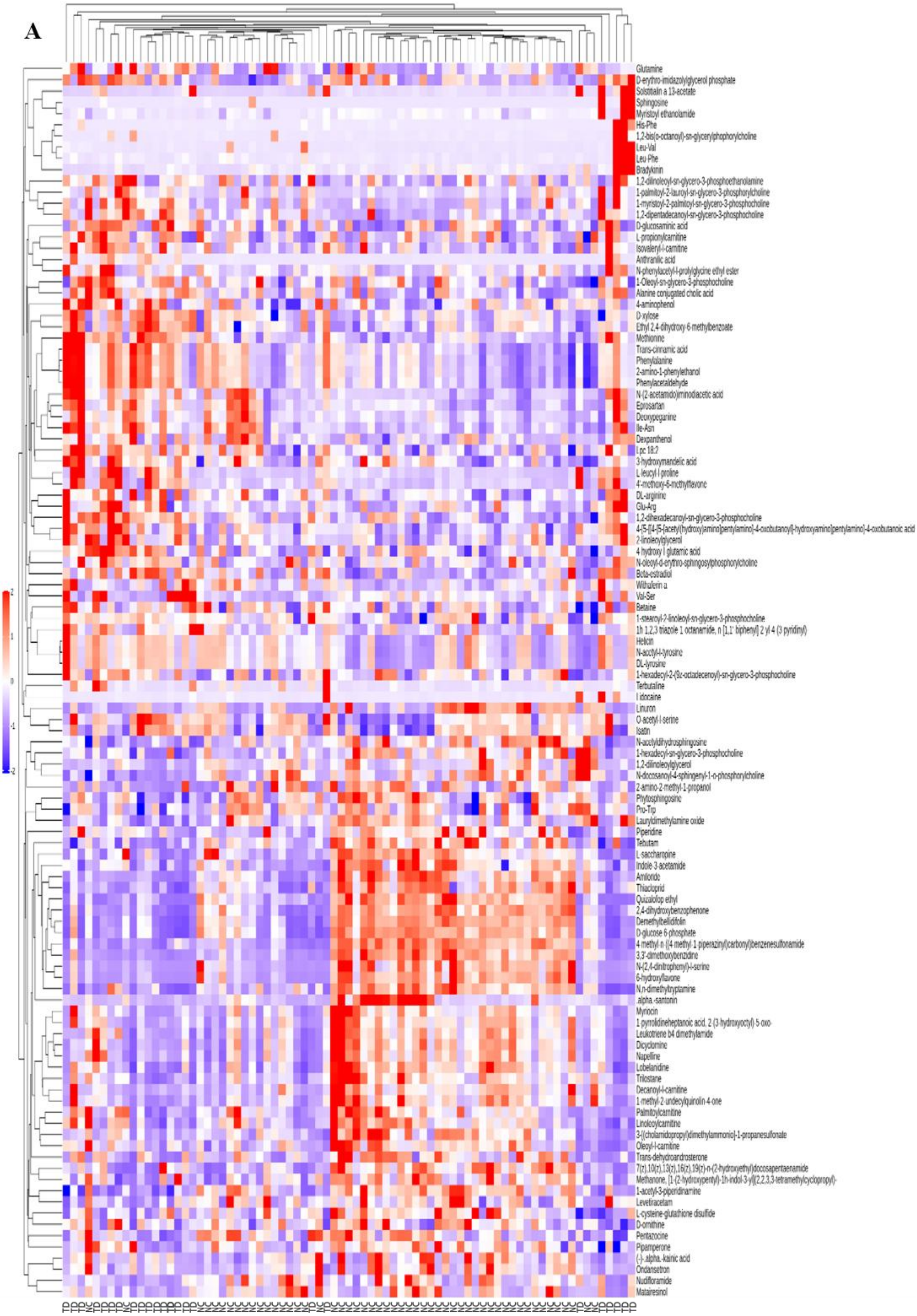

Supplementary Figure 3(A) Hierarchical Clustering Heatmap of significantly differentially expressed metabolites in TD (VIP > 1, p-value < 0.05).

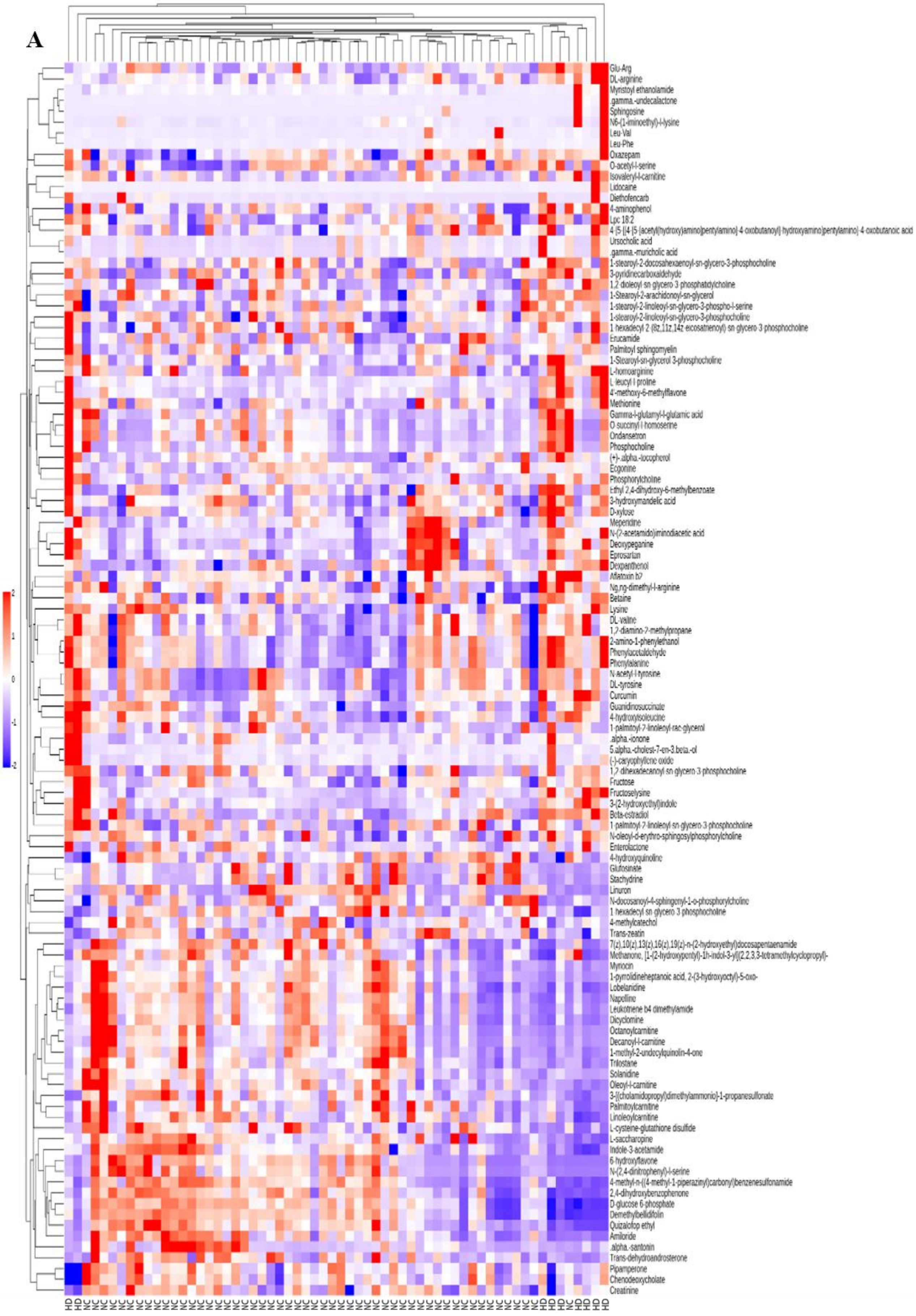

Supplementary Figure 4(A) Hierarchical Clustering Heatmap of significantly differentially expressed metabolites in HD (VIP > 1, *p*-value < 0.05).

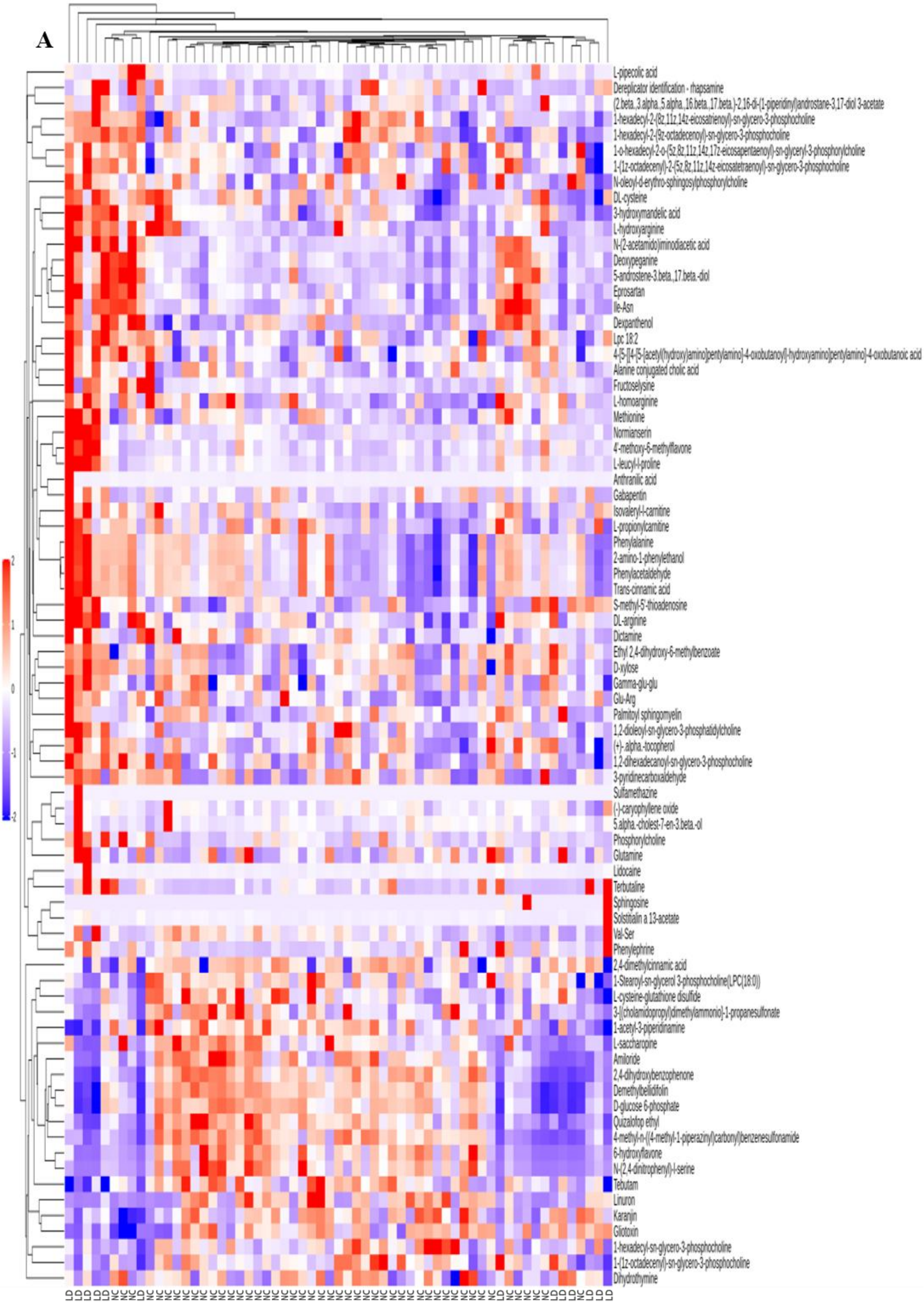

Supplementary Figure 5(A) Hierarchical Clustering Heatmap of significantly differentially expressed metabolites in LD (VIP > 1, p-value < 0.05).

A

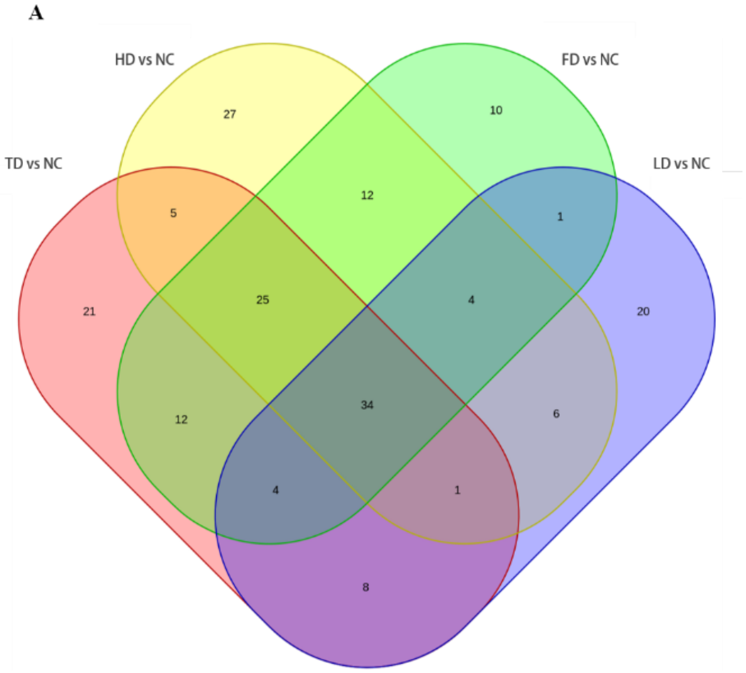

Supplementary Figure 6(A) Venn Graph. In this graph, each circle represents a sample (group). The numbers in the overlapping areas of the circles indicate the number of metabolites shared among the samples (groups), while the numbers in the non-overlapping areas represent the number of unique metabolites in each sample (group). Pink: TD; Yellow: HD; Green: FD; Purple: LD.

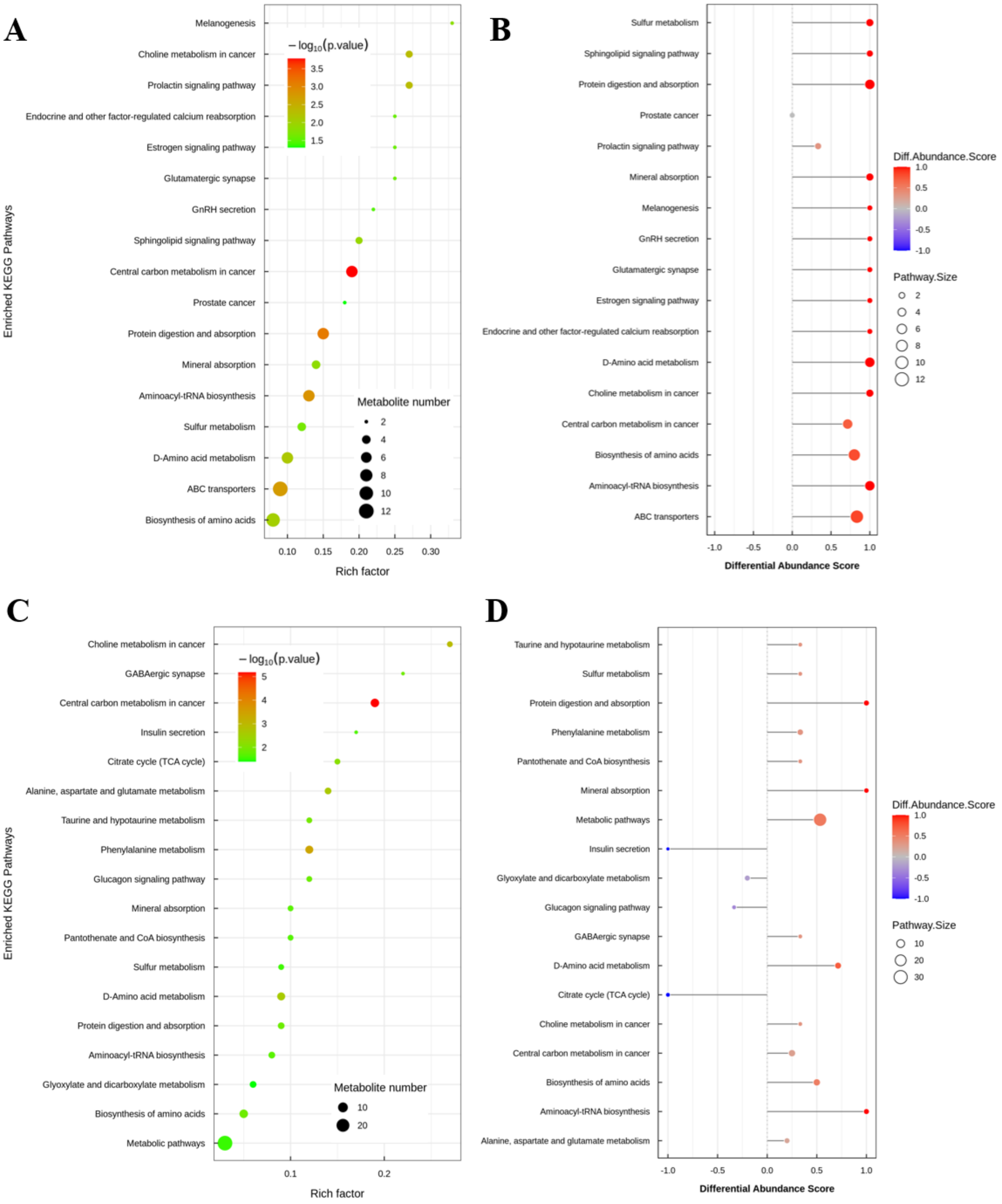

Supplementary Figure 7 (A-B) Bubble plot of KEGG enriched pathways and Differential Abundance Scores plot for HD. (C-D) Bubble plot of KEGG enriched pathways and Differential Abundance Scores plot for LD.

A

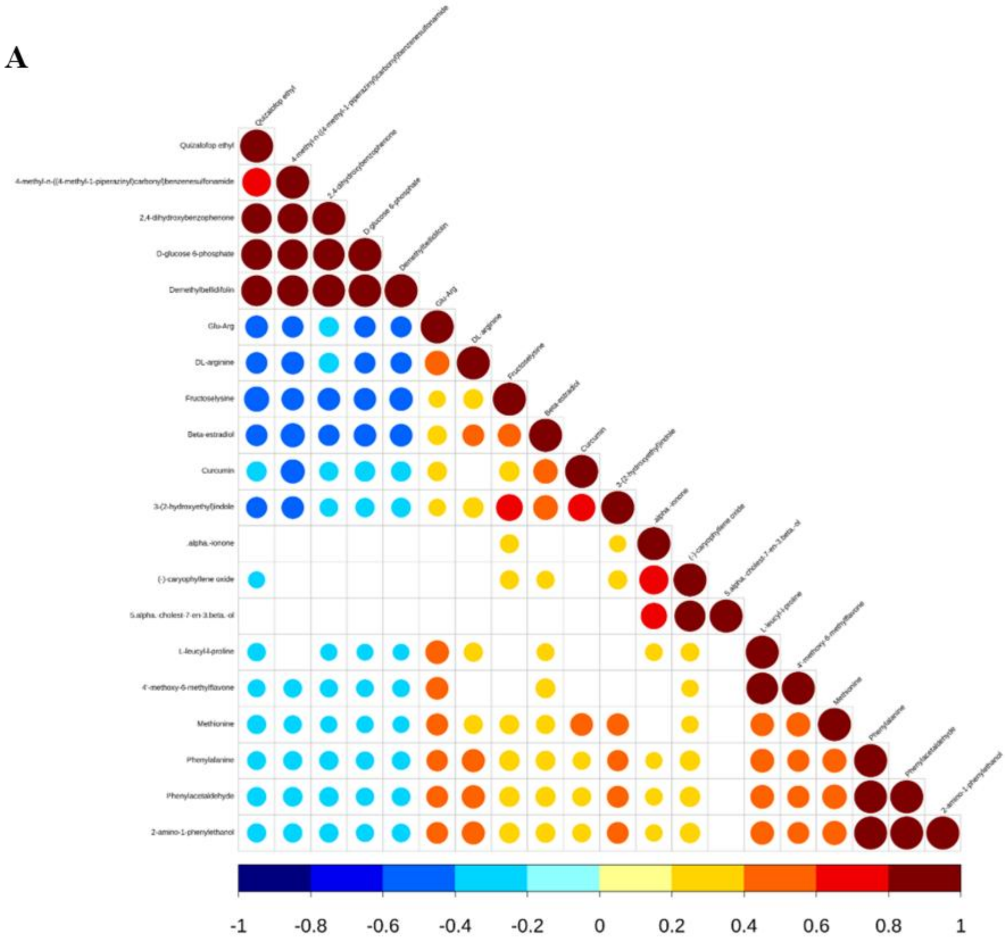

B

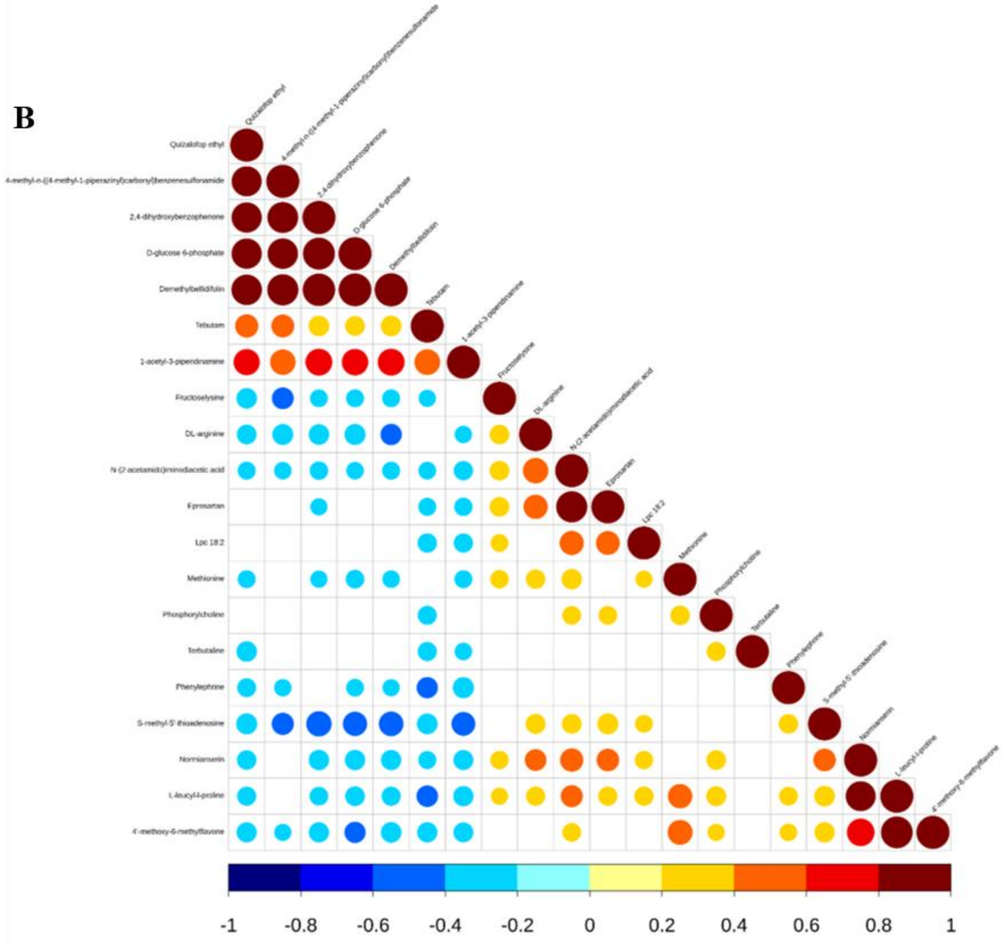

Supplementary Figure 8 Correlation matrix plot of metabolomics. (A) HD; (B) LD.

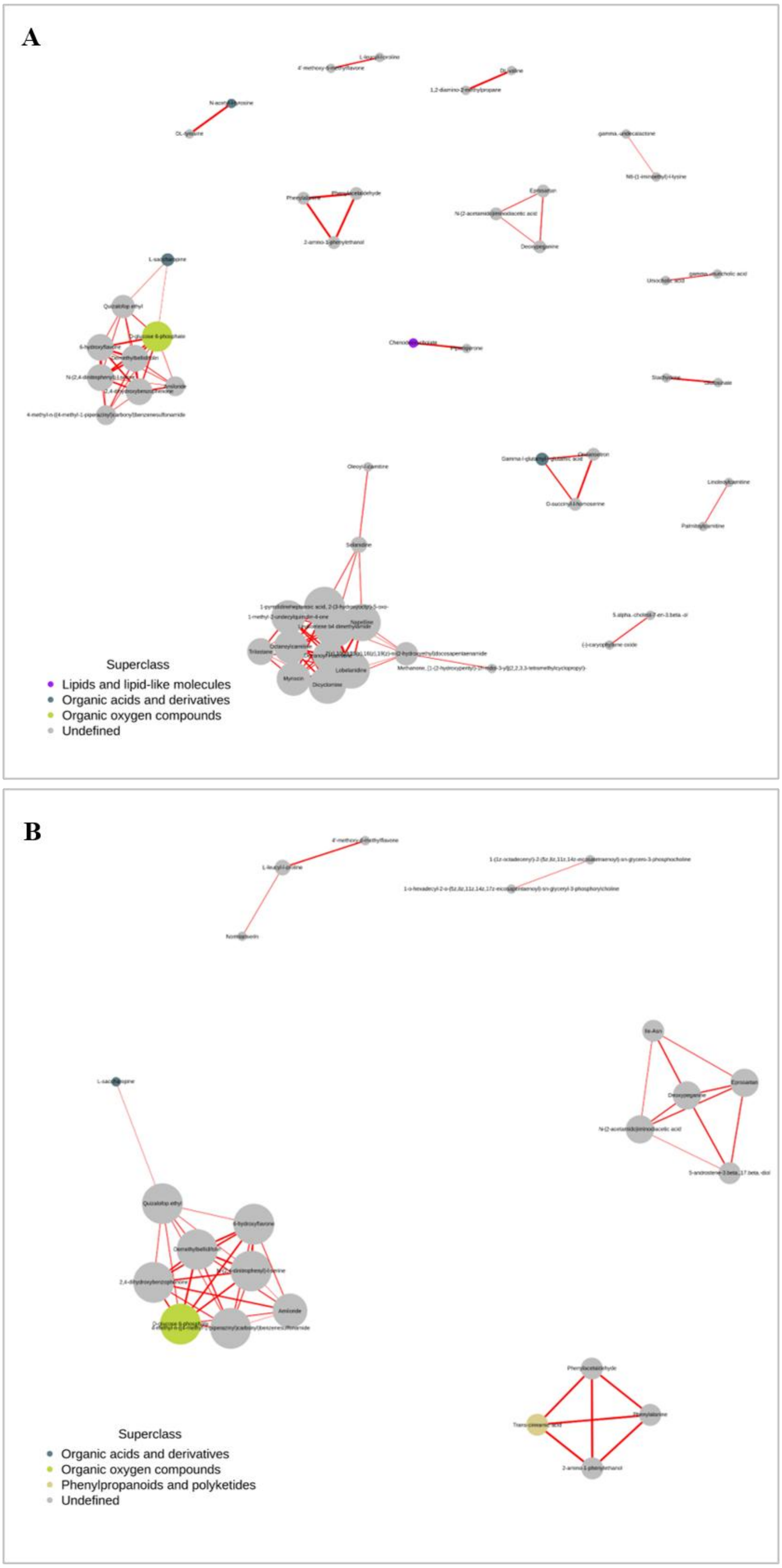

Supplementary Figure 9 (A-B) Network plot in metabolomics for HD and LD.
